# A Memoir on Those Diseases Which Proved so Fatal to Our Troops during the Burman War; with a Comparative Sketch of Their Analogous Cases in Hindostan, during a Service of Some Years

**Published:** 1829-02

**Authors:** James Walsh

**Affiliations:** Assistant Surgeon 89th Regiment.


### DISEASES IN HINDOSTAN.

*A Memoir on those Diseases which proved so fatal to our Troops during the Burman War; with a comparative Sketch of their analogous Cases in Hindostan, during a Service of some years.* By James Walsh, Assistant Surgeon 89th Regiment.

(Continued from p. 35.)

In my own case, which was a secondary intermittent, of that extremely irregular description alluded to, I felt compelled, although still labouring under considerable cerebral and hepatic disturbance, to have recourse to large opiates: otherwise, such was the overpowering depression and indescribable alarm rapidly setting in, that, if not immediately relieved, it must have terminated in death, or worse than death, mental alienation. However, the opiate soon produced a pleasant change of feeling, speedily ending in a sound sleep, with a profuse perspiration, and a consequent solution of the paroxysm. Up to this period, I had always entertained a strong objection to opium, wherever topical affection, even on a small scale, might exist; and in my own person I entered upon it rather against my will, from an early idiosyncrasy, wherein I always found it stimulant and exciting, as well as invariably preventing sleep.

In the course of some years' Indian practice, I yielded rather too much, perhaps, to this prejudice, and may have, one time or other, withheld from my patient no small alleviation of his sufferings, and from myself the satisfaction of witnessing it. However, from the decided benefit following its administration in those intermittents, and other anomalous cases, it is obvious the prejudices against it must have arisen from unfounded theory and causeless apprehension, instead of attentive observation and practical deduction.

Cinchona, next in the scale of exhibition, encountered with me as strong objections as opium ever did, the one for its narcotic properties, and the other for its general inertness. To this conclusion I had long come, from observing its almost total inefficacy in the intermittents of Belgium and North America, where I had seen it rather extensively tried. Whether it might have arisen from a superiority in the quality of the bark used in Pegu, I cannot say, but its beneficial effects were most marked and decisive. I entered upon its prescription for others rather in compliance with general custom or professional routine, than with the hope of advantage: for myself I commenced it merely in obedience to the injunction of a medical friend; but, in a little time, and in a variety of unpromising cases, I found myself agreeably undeceived at every step. These intermittents were not merely obstinate and unmanageable, but extremely apt to return, even after a long interval of freedom: and the relapses not only much more frequent than from similar affections in other countries, but their irregularity and severity were often increased. I have observed the recurrence of ague from a short exposure to a current of cold air after a sudden momentary exertion, or even from a large draught of cold water, when advanced convalescence and returning strength had given hopes of progressive and certain recovery.

Arsenic, I believe, was not often used, nor can I now recollect its exhibition, or even that it was furnished at this period in the list of hospital medicines; but I have elsewhere seen abundant proofs of its general efficacy.

It might be supposed that opportunities of anatomical investigation, so prodigally furnished, ought to have been proportionally attended to; but when it is considered that the hospital was invariably crowded to overflowing, with half, and more frequently three-fourths, of the regiment, all requiring the most minute and repeated attention, it must be admitted that little satisfactory in morbid research could often be obtained.

When we also take into account the officers labouring, at least in equal proportion, as well as under equal or greater violence of disease, with the soldier, and calling as strongly for his share of sympathy and attention, very little time was necessarily left to the surgeon or assistant for his personal wants or attention to his own health, often most seriously impaired. If to this may be added the severe illness of one medical officer, (too often the case,) and the absence of another with a detachment on duty or stockade attack, once or twice a week, and now and then for three or more days successively, it will be considered, perhaps, rather a matter of wonder that an effort at morbid investigation could be made at all. Yet enough was done to satisfy comparative inquiry with their corresponding cases in India, as well as those occurring at subsequent periods, when the force was advancing towards the Burman capital.

The remittent of Pegu was attended with morbid appearances very closely analogous to those of the Indian delta. The effects of cranial determination were sometimes most markedly evident in the increased vascularity and highly turgid state of the vessels, with occasional deposition of lymph or pus on some part of the membranes, and these exhibiting a more or less thickened and altered state. Serous effusion in the ventricles and about the base of the brain has been also observed to a considerable extent. The liver was often found much altered in colour, and so much distended and gorged with blood as scarcely to bear handling or pressure. The gall-bladder now and then nearly empty, or containing a small quantity of thin, light-coloured bile: in other cases it was much enlarged, and, when cut into, discharged a viscid, thick, and dark substance, not unlike tar in consistence or colour. The other organs or viscera evinced little or no trace of diseased action.

This more frequent disorganization of the liver in alluvion remittents, Indian or Burman, affords a contrast to what I have seen in jungle or hill affections, similar in violence or progressive development. The fevers of the pestilential jungle of Wynaad have at times evinced deleterious miasmal influence to as concentrated an extent as the endemic of the west; yet the abdominal viscus most frequently bearing the onus of disease was the spleen, whilst the liver remained uninjured. In the majority of these (Wynaad) cases, the spleen was considerably enlarged, often to twice or three times its ordinary size, and in structure so soft and pulpy as readily to give way on the slightest pressure. It appeared so broken down internally, and so gorged with blood, as to resemble a mass of broken-down coagulum, rather than an organized viscus.

On the other hand, a few of the worst Wynaad cases which I have seen were marked by a singular deviation from the usual morbid results. This description of fever was attended with a highly yellow state of the skin, a like suffusion of the eyes, and generally with excessive gastric irritability and vomiting of matter more or less dark; and, although terminating fatally in four or five days, the traces of local injury or disorganization were so trifling as to excite the highest astonishment at their rapidly destructive results, and that death could have occurred so early without affording any grounds for a just pathological conclusion as to the cause.

The vigorous treatment so imperiously called for to obviate rapid disorganization, must unfortunately, and but too naturally, even when successful, have left the patient in a state of great debility, as well as more susceptible of morbific action than before. Hence, those escaping from the immediate fatality of violent fever were, in general, observed to sink sooner or later under the attack of cholera or dysentery.

Of rheumatism, it was remarked before as being often attended with a vast deal of trouble, but no danger: its treatment, therefore, could have given rise to little embarrassment or anxiety; but in cholera or dysentery there was a miserable reverse, almost every case of the one being carried off with frightful rapidity, whilst those of the latter, although more protracted, were equally fatal in their results.

It is not in the power of language to depict, or scarcely even in a strong imagination to conceive, the state of a large hospital thus circumstanced, crowded to excess with the dead, the dying, and those screaming out, with piercing agony, for relief or dissolution. That medical man must have been made of stern stuff indeed, to be able to look round unmoved upon such a scene as this. Medicine often given up as wholly useless, nearly without assistance or servants, and perhaps also in a state of total want, or at least scanty supply, of cordials, restoratives, or other comforts.

The few who escaped, or evinced any degree of convalescence, were sent to their barracks, as well for change of air and the company and assistance of their comrades, as to afford better accommodation to those remaining in hospital; but the convalescent had soon to be readmitted, sinking under cholera or attacked with dysentery; and it may with truth be affirmed, that almost every man attacked with one or the other, or both, (for they were often combined,) gave himself up for lost, and the prediction was in general too fully verified.

Medicine he ceased to have any faith in, and the medical officer, at times, instead of attending to its exhibition, which the patient despairingly refused, would endeavour to hold out the benefit of resignation and hope, but to very little purpose. The unfortunate soldier would make his will, as he called it, and then lay himself down, sometimes with an appearance of resignation, indeed, but without a ray of hope; at other times with the fixed eye and convulsive shudder of despair.

Of cholera, as it appeared in Burmah, little else might be said beyond the sad record of its attack and fatal result, if the symptoms of this period did not offer a powerful contrast to those occurring when the regiment lay at Madras for ten or twelve days, previous to its embarkation for Rangoon. In those days of health and plethoric fulness, cholera pervaded the corps with epidemic violence and great mortality. The man in his barrack room was as often seized with cholera as his comrade exposed to fatigue and insolation. In the latter case, indeed, the complaint could not early admit of being distinguished, having so mixed a character of high cerebral disturbance and febrile excitement before the more marked spasm of cholera set in. But this made, in the first instance, no difference in the mode of treatment, as the cholera, single and uncombined, of this period (May 1824,) would evince, with the abdominal and spasmodic uneasiness, much vascular and sensorial excitement, calling for bleeding to as great an extent, perhaps, as the synocha itself might require. The other symptoms of spasmodic disease soon appearing, left no room for doubt or much time for remedial effort.

Headache, quick (and in the beginning a strong) pulse, although shortly sinking into collapse, violent spasm more or less diffused, accompanied with incessant gastric and alvine evacuations of a thin watery fluid, suddenly ejected, and often in larger quantity than the fluid administered, would in many cases carry off the patient in a few hours.

The inflammatory action in cholera, although at this time so marked and conspicuous, was not attended with that state of the circulating fluid which takes place in other inflammations. I have bled largely in every case when called on early, and never observed the blood with a buffy surface.

During or soon after the free abstraction of blood, some relief or suspension from gastric irritability would now and then take place, so as to allow the retention of calomel and opium. Time was also afforded for warm baths, with warm spirituous fomentations to the scrobiculus, generally the seat of permanent and intense uneasiness. Constant and unremitting friction with the hand, flannel, hot sand bags, &c. over a great portion of the body, particularly the abdomen, extremities, and other spasmed parts, was often productive at least of partial relief.

Throughout the middle and latter periods of the attack, or growing state of excessive collapse, strong and warm punch, with warm aromatic draughts, to as great an extent as the stomach would admit, were given alternately with calomel and opium. These two powerful articles of the materia medica were in this disease seldom (I might also say never, as far as my observation went,) attended with their usual specific results. Neither stimulus, excitement, nor ptyalism followed, although given to a startling extent, not in grains or drops, but by scruples, drachms, and spoonsful! This conclusion could only be inferred from the recovered cases, of course, which I carefully attended to, and with the utmost anxiety, lest dangerous stupor, coma, or salivation might, after all, snatch my patient away.

The mortality and frequency of the complaint at length became so alarming, that the regiment was embarked and ordered to sea, before quite ready, with the hope of getting beyond its range.

During this short stay at Madras, preparing for the Burman expedition, the deaths may have averaged seven or eight per day, chiefly from cholera. Indeed, such was the violence, rapidity, and frequency of attack, from inflammatory and congestive cholera and bilious synocha, that for five or six days, I might with great truth assert, I had scarcely one hour's unbroken rest or leisure, and that the lancet or prescribing pen had not been out of my hand for several succeeding hours together.

A great number would appear to have carried the *materies morbi* with them on board, as about twenty-five out of two hundred in a transport under my charge were attacked with cholera of the same kind, in the course of the voyage. Of these, three died; the remainder became quickly convalescent, notwithstanding the debilitating and other effects of profuse depletion, opium, and calomel.

For the first few days on board, my difficulties were rather appalling: labouring under illness myself, severe enough, I thought, to render me incapable of exertion, and without the assistance so abundantly procurable in India under other circumstances; as the servants engaged, and unfortunately paid too largely in advance, had all ran away at the moment of embarkation. Thus situated, I had to request the assistance of such of the soldiers as could be induced to volunteer for hospital duties; duties which, in India, Europeans are seldom or never called on to perform, and which, of course, they had the greatest repugnance to. This feeling of repugnance became soon in a great measure insurmountable, from a dread of infection having taken possession of their minds, and it must be admitted with, to them, some show of reason, as three of the men, some days on board, and up to this time in apparent good health, were soon seized with cholera, as they were attending to the wants of their comrades sinking under the same complaint. One of these orderlies quickly became a martyr, as they considered then, in the execution of the duty imposed on him, but to which he devoted himself with energy and perseverance: the two others had nearly shared the same fate.

During this period of apprehended infection, I had to remain in the hospital birth several hours each day, for some days, before I could divest those about me of their unfounded fears. However, my perfect immunity and confidence soon excited a similarity of feeling on their parts, and with flattering results, as I did not lose another man for the remainder of the voyage.

This sketch of cholera, as met with occasionally, not only at Madras, but along the Malabar and Coromandel coasts, as well as throughout a large portion of the Madras presidency which I have traversed, is the result of as much attentive observation and comparison as I could have exerted. I set myself down to the contemplation of this most dreadful scourge with a mind as much divested of preconceived theory and bias as could well have been effected under the great variety of circumstances presenting themselves, but still, with all this aptitude for free inquiry, I observed little or nothing for a satisfactory conclusion, unless that many, if not most, of the alleged exciting causes had no foundation in fact.

It has been said to occur most frequently at night; to have arisen from suppressed perspiration, or a rapid transition from a high to a lower range of temperature; whereas the opposite in all was as often the case.

The absence or presence of the sun evinced little influence, at least as an immediately exciting cause, the attacks being, of the two periods, more frequent during the day than at night. Still less could it be said to have arisen from suppressed perspiration, or atmospheric vicissitude, as the men, off duty for days, and in their barrack rooms, would have had little occasion for exposure or exertion that could induce dangerous perspiration, yet these men were affected to as great an extent as the others.

Neither age, sex, nor other personal circumstance, seemed productive of any peculiar exemption. The child, the man, or the woman, of any colour or age, were alike susceptible of its violence.

I have known the child, within doors, attacked at the same time and to an equal degree with her father, who was absent on business six or eight hundred yards; and the sympathy, during recovery, which took place in both, was as remarkable as at the beginning of the complaint. The father had been away for hours, of course, may be said to have been more or less exposed; but the child, who became ill some time before his arrival, but precisely at the same period of attack, as subsequently ascertained, was coolly and quietly amusing herself in a large and comfortable room, with the advantage of being open to the seabreeze.

Indian cholera having been so ably treated of by others, and with such superior research and accuracy as to leave little original or new for my limited range of inquiry, would perhaps not have been entered upon at all, but for comparative effect with the mortality of Burmah. In the epidemic cholera of India, as affecting Europeans in good or tolerable health, the cerebral disturbance, unequal nervous excitement, and consequent reaction, would lead to the inference that the cranial contents had sustained the first shock of disease, to which a new or different mode of action from that in the bilious remittent is soon superinduced.

The distribution of the nervous energy becomes unequal and irregular. The muscles are thrown, more or less, into a state of spasm, communicating a like irregularity to the circulating fluid, soon becoming in itself the cause of more complicated mischief. Increased determination of blood to the head, as well as cerebral nervous excitement, must produce corresponding derangement and congestion in other parts, particularly those with a complex vascular structure and a secreting function. Wherefore it is that the head, stomach, and liver are in a state of such functional disturbance and unequal derangement: still these organs very seldom exhibit, in the most violent cases, any sufficient appearance of morbid structural injury to account for the fatal result.

In the Burman cholera, on the other hand, a tendency to cerebral disturbance, increased vascular action, or hepatic congestion, was scarcely ever to be observed. The *vis vitæ* was almost invariably so overpowered, that reaction could not be brought about by any effort of medicinal stimulus, or relief from spasm by opiates or friction to any extent. Indeed, in the immense majority of these cases, the state of collapse and progress to dissolution were so great, that the patient would appear *in articulo mortis* before he could be well received in hospital; and if the spasm and extreme collapse did not at the time give to this destructive malady its peculiar characteristics, the nosologist might feel at a loss what class or order to place it in.

Although in India little certain, beyond terrestrial exhalation and a particular state of the atmosphere, can be set down as the proximate or remote sources of cholera; in Burmah, extreme debility, or great exhaustion, would appear to be the immediate causes, to which atmospheric morbific impregnation might have superadded a specific and fatal action. But we can be at no loss for remote or predisposing causes, when we recur to the catalogue of extreme fatigue, incessant exposure, and an hitherto unheard-of privation, to which the soldier was for a time subjected, and of which an imperfect outline was given before. To these may be added the debilitating effects of the febrile epidemic pervading the whole force in the first instance, so fatal to many, and necessarily so productive of an excessively morbid disposition in those who survived its attack.

Dysentery, of the most irremediable description, next presented itself, sweeping off the greater part of those who survived the prior forms of disease, as well as extending its baneful visitation to those comparative few who had been hitherto exempt from illness. In tracing its causes, we can have little occasion to go beyond the exposure and fatigue already pointed out, and the state of inanition which the soldier would sometimes bear to a certain extent, sooner than have recourse to the highly unsound provisions at this time served out. It would, indeed, be rather inconsistent with facts founded on such privation and suffering, to lay down repletion as in the remotest degree auxiliary to morbid agency. Where the rations were not merely insufficient, but for a long period so bad as to be wholly incapable of affording either sustenance or stimulus to the man called upon, to such an unprecedented degree, for exertion and energy. Those rations alone might, therefore, be set down as chief and foremost in the list of predisponent causes; as, although so little capable of nutriment, yet their morbid action on the intestinal canal must be in an inverse ratio to the little they possessed.

Without seeking in any peculiar state of the atmosphere as an exciting or predisponent cause of this unusual form of dysentery, it will, perhaps, be admitted that enough of tangible grounds are furnished in this slight detail. We can be, therefore, at little loss to account for the low state of general excitement, the absence of inflammatory action, the accompanying pyrexia, resembling more the typhoid type, or rather what would have marked the scorbutic state of the system as fully developed.

This appearance of scurvy gave a peculiar character to the disease in almost every stage of its progress. The turgid, livid, and fetid state of the gums; the countenance, whether bloated or sunken, exhibiting depraved habit of body; the ulcerated or blotched state of the legs and other parts; with the early loss of strength, incapability for exertion, and great mental depression, afford a tolerable certainty of this state of the system; and, combined as it was with severe dysentery, made it no less unmanageable than hopeless. It would, therefore, rarely admit of depletion, notwithstanding great abdominal uneasiness with tension, as also tormina, tenesmus, and discharge of blood: in fact, wherever it was practised, the fatal catastrophe seemed to have been hurried on. Mercury, too, in a short time was obliged to be discontinued, as, although it might, for this short period, in some cases produce a temporary relief from the more urgent symptoms, yet, as soon as its proper action would appear, the whole train of symptoms, scorbutic and dysenteric, often underwent such increased exasperation as not to admit of its further exhibition. Our treatment, therefore, was limited to palliatives in every possible form; such as fomentations, topical bleedings, mild purgatives, anodynes, blisters, frequent opiate and other enemas, &c.

Cholera, in a state of collapse, would sometimes show itself in this form of dysentery, and in general quickly terminate the patient's sufferings.

Now and then a solitary case has occurred where the collapse was successfully resisted by active stimuli, at first rather despairingly ventured on, in consequence of the preexisting abdominal irritation; and yet in this latter relief was had also to an unexpected extent; so that wine, opium, and diluted spirit, were afterwards freely administered in this alone, and with obvious benefit, but, alas, too often transient and illusory.

Mercury, having been found to exasperate all the symptoms, both scorbutic and intestinal, was generally laid aside, and an attempt made to improve the scorbutic state of the system merely by regimen and diet; at least, such a course as our situation would admit of procuring. Light and nutritious broths and jellies, animal and vegetable, were given in every form that could gratify the feelings of the patient. Vegetables and fruits, (plantains, pineapples, and limes, the only procurable ones,) were also tried, but at length obliged to be discontinued; as, although the symptoms more purely scorbutic would seem to have improved, the dysentery was increased, and rapidly advancing to a fatal conclusion: yet this was suspended in a few cases by giving six or twelve ounces of wine per day, with twelve or sixteen grains Pulv. Ipecac. compos. twice or three times in the same period, according to the urgency of the symptoms; or two pills every third hour, ex Opii gr. $\frac{1}{2}$, Pulv. Ipecac., Pilul. Hydr. *aa* gr. ij. M. singula. The small quantity of bluepill, thus combined, was seldom attended with any mercurial effect, but would seem to be a useful adjuvant in this soothing, and by the patient much-wished-for, medicine; being in general found the best calculated for smoothing the unhappy man's passage to the grave, as a probable escape from it he had early taught his mind not to entertain the most distant hope of.

The symptoms in this combination of disease, or scorbuto-dysentery, were, *ab initio*, tolerably marked; as were also their progressive accompaniment and mutual reaction, with, of course, their consequent exasperation. Still the disease rarely proceeded to a fatal termination with that rapidity which so strongly characterizes the idiopathic form of Indian dysentery, yet with much greater certainty.

That speedy tendency to organic lesion which takes place in the one, unless counteracted by profuse bleeding, mercurials, &c. would appear to be retarded in the other (where these remedial means are inadmissible) by the extraordinary modification impressed upon it, or arising from, the scorbutic action on the system. This diathesis, giving rise to more or less prostration of strength, diminished nervous and vascular energy, the remoter, although more certain, destruction of the organ or viscus particularly affected, may be thus accounted for.

In two or three cases only the scorbutic action evinced any fatal predominance; one more particularly in the Tavoy anchorage, September 1824.

In this case, the usual symptoms of both diseases were at first distinctly marked, but in a few days the gums evinced a high degree of fetor and virulent ulceration. The ulcerative process soon put on the most malignant and phagedenic form. The gums, lips, and tongue were attacked with extraordinary rapidity; the entire flesh and integuments of the lower jaw and contiguous portions of the neck, with the tongue enormously swoln and protruded, were all soon involved, as it were, in the highest and most destructive species of sphacelation, presenting the most hideous appearance that can well be imagined. As this man was one of the comparative few who hitherto escaped illness, and being apparently plethoric, with a robust form, although with rather a feeble pulse, was bled largely, i.e. *ad deliquium,* on the first day of the complaint assuming this destructive character, but without the slightest benefit, as it, on the contrary, evinced greater violence. Acids, opium, bark, wine, antiseptic applications, &c. in every variety of form, were then had recourse to, but ineffectually, as he died early on the fourth day. Yet, notwithstanding the most careful examination, we could not find, in the post-mortem appearances, any thing to account for this singularly rapid and horrible result.

The intestines evinced a good deal of vascularity, with flatulent distention, and incipient lividity in the peritoneum; but this could not in any degree have added to the frightful destruction about the mouth. The pharynx, œsophagus, and larynx were unaltered, except in the immediate neighbourhood of the morbid action, where a limited redness would seem to mark the approach, perhaps, to a line of separation, if the profuse hemorrhage and excessive prostration of strength had not speedily overwhelmed the patient. The corrosive action was so great as soon to involve every thing in its ravages, skin, muscles, tendons, cartilage, blood-vessels, the periosteum, and to a certain extent the bones themselves; three or four teeth, with their alveoli and part of either maxilla, were almost coughed out at two periods, when convulsively expectorating the blackish, broken down, putrid mass, in which no distinction of parts whatever could be observed.

This terrific affection, resembling so much the ravages of hospital gangrene, would have filled me with the greatest dread lest it might propagate itself, in the supposed contagious manner of that disease, being then on board a crowded hospital ship, with men labouring under scorbutic dysentery, cholera, and other complaints equally asthenic; of course, affording a ready and extensive field for infection. But I had long before divested my mind, in a very great degree, of the existence of any such morbific power as tropical contagion; and in this case no such agency discovered itself, occurring singly, and fortunately terminating so.

Notwithstanding this case of violent and destructive action, arising evidently from a scorbutic diathesis, the dysentery of this form was not in general rapid in its progress, although so unmanageable and almost invariably so fatal; yet the post-mortem appearances, in most cases, would mark very severe disease.

The peritoneum lining the abdominal cavity was in general highly vascular, with some dark and livid patches interspersed. The livor in some had amounted to gangrene; in others, ulceration and deposition of lymph and pus had partially taken place. The hepatic organs in general were but little affected; at least, not evincing a primary diseased action in this complaint. The spleen was sometimes enlarged and morbidly soft. The stomach and duodenum but little altered on their internal surface, while the peritoneal coat presented appearances similar to that lining the abdominal parietes. The intestines exhibited the onus of disease most severely, in fact, nearly throughout their entire canal, from the duodenum to the rectum. The canal was in many places thickened and contracted; in others, so thinned and distended as to rupture with the slightest handling. The greater portion of their peritoneal coat was livid and gangrenous, the sphacelus extending at many places through all their coats. In other portions, ulceration would appear to have made its way from within, often to a great extent, and in some places might have admitted of the passage of their fecal contents into the abdominal cavity.

Throughout a considerable portion of the jejunum, ileum, and large intestines, the mucous membrane was much abraded. This abrasion extended itself from the various points of ulceration, and was now and then covered with layers of coagulable lymph, adding to its apparent thickness, but so little organized as not to bear the slightest pressure or distention. It was, indeed, a matter of great surprise how the peristaltic motion could have been so long borne by the intestines in this state of disorganization.

Their fecal contents exhibited great diversity of colour, consistence, and odour. Their colour alternating between a yellow, dark greenish, or lateritious brown; more frequently thin, abundant, and sometimes with shreds of abraded membrane to an unusual extent, but never containing scybalæ. The odour always highly fetid and extremely disagreeable. Pus, mucus, and blood were now and then passed in large quantity during the latter stages of the complaint, and, after death, often appeared in the intestines in a separate state.

During convalescence, the progress towards recovery was always slow, and now and then interrupted by a relapse. An apparently trifling deviation in an ordinary article of diet, such even as eating a plantain or piece of pineapple, has reproduced it.

In those cases of occasional relapse and ultimate recovery, the complaint would gradually assume the state of chronic diarrhœa, with evacuations rather frequent, uniform, and now and then consistent, without blood or mucus. The evacuations continued for a long period, eight or twelve months, or more, of a dark brown colour; scarcely ever, for this time, evincing any marked change, although some improvement and variety of diet may have been obtained. The scorbutic symptoms were the first to subside, the gums becoming healthy and clean, the cuticular affections gradually disappearing, with that renovated state of mental sensation indicative of an approach to elasticity and vigor.

[To be continued.]

---

